# Editorial: Immune Complexes in Disease Pathology

**DOI:** 10.3389/fimmu.2017.00173

**Published:** 2017-02-20

**Authors:** Anil K. Chauhan

**Affiliations:** ^1^Internal Medicine, Division of Rheumatology, Saint Louis University, School of Medicine, Saint Louis, MO, USA

**Keywords:** immune complex, systemic lupus erythematosus, membranous nephropathy, syk inhibitor, lung diseases

**Editorial on the Research Topic**

**Molecular Mechanisms of Immune Complex Pathophysiology**

Foreign and modified self-antigens bind to natural IgM to form immune complexes (ICs). This facilitates the instructive phase of immunity, which programs immune response to generate class-switched high affinity antibodies required for IC formation, resulting in pathogen clearance. ICs are the first responders of immune challenge. However, accumulation of abnormal IC levels triggers disease pathologies such as nephropathies, autoimmunity, cancers, infections, and lung injury. ICs are dynamic in their size and composition, present either in systemic circulation or formed locally in tissue. In seven reviews in the topic “Molecular Mechanisms of Immune Complex Physiology,” the authors describe various pathological and physiological aspects of IC-driven pathologies. Krishna and Nadler summarize the consequence of ICs formed by biologics and their role in antigenicity to biotherapeutics. Knoppova et al. summarize how galactose-deficient IgA1 bound to IgG or IgA, upon forming ICs deposits in glomeruli activate mesangial cells, induce renal injury, and trigger cellular proliferation. In membranous nephropathy, autoantibodies to phospholipase A2 receptor expressed by podocytes form ICs *in situ*. Borza discusses how renal injury results from dysregulation of the alternate pathway of complement due to loss of heparin sulfate chains. Classical complement pathway activation by ICs results in the production of C5a, a potent anaphylatoxin, and membrane attack complex (MAC or C5b-9). Sublytic C5b-9 triggers signaling and induces growth factors and cytokine secretion in various cell types. C5b-9 in CD4^+^ T-cells triggers membrane rafts (MRs) clustering, a function attributed to CD28 cosignaling. MRs are membrane organelles rich in signaling proteins that play a crucial role in lymphocyte activation. Ward et al. describe in their mini-review how ICs participate in the development of acute respiratory distress syndrome and acute lung injury. These authors further describe how generation of C5a by ICs upon binding to *C5aR1/C5aR2* promotes the appearance of extracellular histones, which are a major component of neutrophil extracellular traps (NETs). In autoimmunity, ribonucleoprotein-containing ICs induce NETosis. It is now proposed that the histone citrullination and reactive oxygen species are important for NETosis (1). Juvenile idiopathic arthritis (JIA) is characterized by the presence of elevated ICs, and IC deposits are observed in patient joints. Moore reviews the presence of ICs in JIA and discusses the role of citrullinated peptide antigens such as type II collagen and fibrinogen, which form ICs that contribute to disease pathology. Low-affinity Fc receptors bind to ICs and signal by phosphorylating Syk, which then utilizes PLC-γ2 downstream. These events have been well studied in B cells. Mutation in gene encoding for ZAP-70, a tyrosine kinase protein known to play a key role in T cell receptor (TCR) signaling, diverts signaling to Syk, and this shift is associated with the development of autoimmune response. Syk signaling is also observed when membrane toll-like receptor (TLR) 4 recognizes LPS. The mini-review by Deng et al. summarizes the downstream signaling events associated with Syk phosphorylation. These authors further discuss the role of Syk activation in various autoimmune modalities and the development of Syk inhibitors for therapeutic use. Signaling from TLRs, which mediates the innate immune response, has gained prominence due to its role in nucleic acid sensing. In systemic autoimmunity, DNA or RNA-containing ICs (DNA/RNA-ICs) act as vehicles to deliver nucleic acids to endosomes, where they are recognized by nucleic acid-sensing TLRs (NA-TLRs). In particular, TLR9 triggers production of type I interferon (IFN) and induces IFN-stimulated gene expression. Production of IFNs by plasmacytoid dendritic cells (pDCs) drives systemic autoimmune responses. In pDCs, FcγRIIa delivers DNA/RNA-ICs to the endosomal compartment. In a review article on this topic, I summarize the past literature on FcRs’ expression in human CD4^+^ T-cells Chauhan. In this review, I propose a model of a possible cross talk between FcRs–TLRs–TCR, which in the germinal center could drive the differentiation of naïve CD4^+^ T-cells, resulting in B-cell help from the generation of follicular helper T-cells, which contribute to autoantibody production. FcγRIIIa signaling in naïve CD4^+^ T-cells leads to the development of proinflammatory effector T-cells (2). FcγRIIIa signaling induces expression of NA-TLRs, which then localize on the cell surface with FcγRIIIIa (2). FcγRIIIa in CD4^+^ T-cells, upon binding to DNA/RNA-ICs, will deliver nucleic acids to endosomal NA-TLRs resulting in a type I IFN response. NETs also release DNA, and nucleic acid sensing from this DNA release will trigger immune response *via* TLR9 in CD4^+^ T-cells. FcγRIIIa signaling drives the accumulation of TLR9 as a full-length protein on the cell surface, where it recognizes CpG ODN 2006. TLR9 on cell surface is critical in discriminating modified self-DNA from encapsulated viral-DNA. In summary, these review articles highlight recent progress and propose new mechanisms to explain how ICs can drive pathology in lung, kidney, and autoimmune disorders.

## Author Contributions

The author confirms being the sole contributor of this work and approved it for publication.

## Conflict of Interest Statement

The author declares that the research was conducted in the absence of any commercial or financial relationships that could be construed as a potential conflict of interest.
